# Depression, anxiety, and psychosocial stressors across BMI classes: A Norwegian population study - The HUNT Study

**DOI:** 10.3389/fendo.2022.886148

**Published:** 2022-08-10

**Authors:** Trine Tetlie Eik-Nes, Audrey Tokatlian, Jayanthi Raman, Dean Spirou, Kirsti Kvaløy

**Affiliations:** ^1^ Department of Neuromedicine and Movement Science, Norwegian University of Science and Technology (NTNU),, Trondheim, Norway; ^2^ Stjørdal Community Mental Health Centre, Department of Psychiatry, Levanger Hospital, Nord-Trøndelag Hospital Trust, Levanger, Norway; ^3^ Discipline of Psychological Science, Australian College of Applied Professions, Sydney, NSW, Australia; ^4^ School of Psychological Sciences, University of Newcastle, Sydney, NSW, Australia; ^5^ Discipline of Clinical Psychology, Graduate School of Health, University of Technology Sydney, Sydney, NSW, Australia; ^6^ Department of Endocrinology and Diabetes, Blacktown Hospital, Sydney, NSW, Australia; ^7^ School of Medicine, Western Sydney University, Sydney, NSW, Australia; ^8^ Department of Research and Development, Levanger Hospital, Nord-Trøndelag Hospital Trust, Levanger, Norway; ^9^ Health Study of Trøndelag (HUNT) Research Centre Department of Public Health and Nursing, Faculty of Medicine and Health Sciences, Norwegian University of Science and Technology (NTNU), Levanger, Norway; ^10^ Centre for Sami Health Research, Department of Community Medicine, Faculty of Health Sciences, Arctic University of Norway (UiT), Tromsø, Norway

**Keywords:** depression, anxiety, psychosocial, stressors, obesity, epidemiology, trauma

## Abstract

**Background:**

Obesity is a global issue with detrimental health impacts. Recent research has highlighted the complexity of obesity due to its psychological correlates. The purpose of the present study was to explore the relationship between body mass index (BMI) and depression, anxiety, and psychosocial stress.

**Methods:**

Data, including demographic, height, and weight information from 23 557 adult participants was obtained from the fourth survey of the Norwegian population based Trøndelag Health Study (HUNT4, 2017-2019). The Hospital Anxiety and Depression Scale (HADS) was used to measure self-reported depression and anxiety. We also collected data on 10 domains of psychosocial stress (violence, mental violence, unwanted sex, cyber bullying, school bullying, history of own life-threatening disease, life-threatening disease in family, relationship problems, divorce, and sudden family death), which were aggregated into a cumulative measure of psychosocial stress.

**Results:**

Multinomial logistic regression was utilized for statistical analysis. In the full model, the relationship between depression, anxiety, and psychosocial stress were explored controlling for age, sex, income, marital status, and educational attainment. After adjustments, a significant relationship was found between depression and obesity I (OR = 1.05, 95% CI 1.03-1.06, *p* <.001) and II and III (OR = 1.10, 95% CI 1.06-1.14, *p* <.001). After the same adjustments, significant relationship between anxiety and overweight and obesity class I was found among elderly participants (≥65 years old). Psychosocial stress significantly and positively related to all levels of BMI, with or without considering anxiety and depression, after controlling for sex, age, educational attainment, marital status, and income in all age groups.

**Conclusions:**

Obesity is a multifaceted health problem, significantly related to psychological factors including depression and psychosocial stress, which supports the need for a multifaceted, targeted approach to obesity treatment.

## 1 Introduction

Obesity is a global issue with detrimental health and economic impacts (1), whereof health consequences include increased risk of cancers, cardiovascular disease, and type 2 diabetes (2, 3). Such health consequences place a substantial direct and indirect burden on health systems and cost an estimated 2 trillion US dollars worldwide per annum (4). In Norway, results from the HUNT4 Survey (2017–2019), indicate that the prevalence of overweight and obesity among adults are 46% and 23%, respectively (5). As the rate of obesity escalates, the burden on available resources is expected to increase, requiring urgent intervention to manage the prevalence of obesity (4).

Conventional treatment of obesity has largely focused on short-term weight modification, with limited emphasis on the factors affecting longer-term weight maintenance (6) such as psychological factors. However, psychological factors often co-occur with obesity and may consequently maintain obesity (7). Although psychological factors such as depression, anxiety, and psychosocial stress have been shown to be associated with obesity (8–10), more research is required to clarify and elucidate how these psychological mechanisms relate to obesity.

Depression is a highly prevalent mental health disorder that has been found to be associated with obesity (10) across epidemiological, clinical, cross-sectional, and longitudinal studies (10–12). Researchers have theorised that this association may be due to increased weight stigma, social discrimination, and eating for the purpose of emotional regulation (13–15). Further, a bidirectional relationship between depression and obesity has also been suggested, where increasing severity of depression leads to an increase in body mass index (BMI) and increased BMI leads to an increase in severity of depression (12). Although a positive association between obesity and depression has been consistently found, sex and educational attainment may influence this relationship (16). For example, previous research has shown a negative association between depression and BMI in a low socioeconomic sample (16). Therefore, a comprehensive understanding of the relationship between depression and BMI should be further explored, taking into consideration potential mediating variables.

Anxiety is another major psychological disorder associated with obesity (9). The relationship between anxiety and BMI, however, appears to be inconsistent across the literature. Systematic reviews have demonstrated a moderate, positive relationship between anxiety severity and BMI (9, 17). In addition, while some findings indicate a non-linear U-shape association (18), others suggest the opposite, an inverted U-shape (19). As a result, there is evidence to suggest that anxiety is high at underweight, low at overweight, and then high again at obesity (20). Conversely, there is also evidence to indicate that anxiety is low at underweight, high at overweight, and low again at obesity (19). One reason for these differences may be confounding variables such as ethnicity, age and socioeconomic status. For example, while an inverse U-shape association has been shown in aged individuals (18), a U-shape association has been found in Caucasians and people of African descent, with no association in Asian or Hispanic populations (18). Moreover, as anxiety and depression are highly prevalent and linked with obesity, exploring their association is essential for building a clearer understanding of the multifaceted nature of obesity.

In addition to depression and anxiety, psychosocial stress may have important implications on obesity. For example, high levels of stress may change eating patterns (21) and may lead to excessive food intake toward a higher fat and sugar diet (22). Allostatic load, described as chronic exposure to stressors, has also been shown to reduce response and adaptation to stress (23). Importantly, a strong correlation between obesity and allostatic load has been found (24), highlighting the importance of further understanding the role of chronic stress in obesity.

Further, a history of trauma both in childhood and adulthood has been demonstrated to have an association with obesity (25, 26). Trauma refers to events that are perceived as sudden, uncontrollable, extremely negative, and continue to negatively impact individuals into the future (27). Hence, traumatic life events are associated with poorer physical and mental health, poverty, lower educational attainment, and obesity (28–32). With regards to obesity, traumatic events may dysregulate the body’s stress response system (i.e., hypothalamic-pituitary-adrenal [HPA] axis (33), resulting in a greater need to emotionally regulate. To reduce the stress-induced activation of the HPA axis, individuals exposed to traumatic life events may consume food high in sugar and fat through the form of disordered eating behaviours such as binge eating (34), which may contribute to weight gain (34, 35). Together, these processes may help to understand the relationship between psychosocial stress and obesity. However, as psychosocial stress is associated with several adverse outcomes including mental health difficulties, it is important to explore whether an independent relationship between psychosocial stress and BMI exists, controlling for depression, anxiety, age, sex, and educational attainment.

Furthermore, sex, educational attainment, and socioeconomic status have been found to relate to obesity, and may mediate the relationship between BMI, depression, anxiety, and psychosocial stress (36). Research has shown that individuals with lower education are more likely to have obesity compared to individuals with higher educational attainment (37). Married individuals are also more likely to have obesity than single individuals (38). Moreover, although there are mixed findings regarding sex differences (39), lower socioeconomic status has been linked with higher obesity levels (40). Therefore, it is important that these potentially mediating variables are accounted for when investigating the relationship between obesity and psychological factors.

The purpose of this study was to explore how depression, anxiety and psychosocial stress independently relate to BMI across four weight categories, including (1) normal weight (2), overweight (3), obesity class I, and (4) obesity classes II and III. As these psychological factors are likely to be mediated by other variables, we explored how they may relate to BMI independent or dependent of each other, controlling for age, sex, income, marital status, and educational attainment. Obesity classes II and III were combined for brevity (41) and the underweight group was not included as there were too few in this category in our sample. We hypothesised that as depression score, anxiety score and psychosocial stressors increase, so will the BMI class.

## 2 Method

### 2.1 Participants

**Figure 1:**
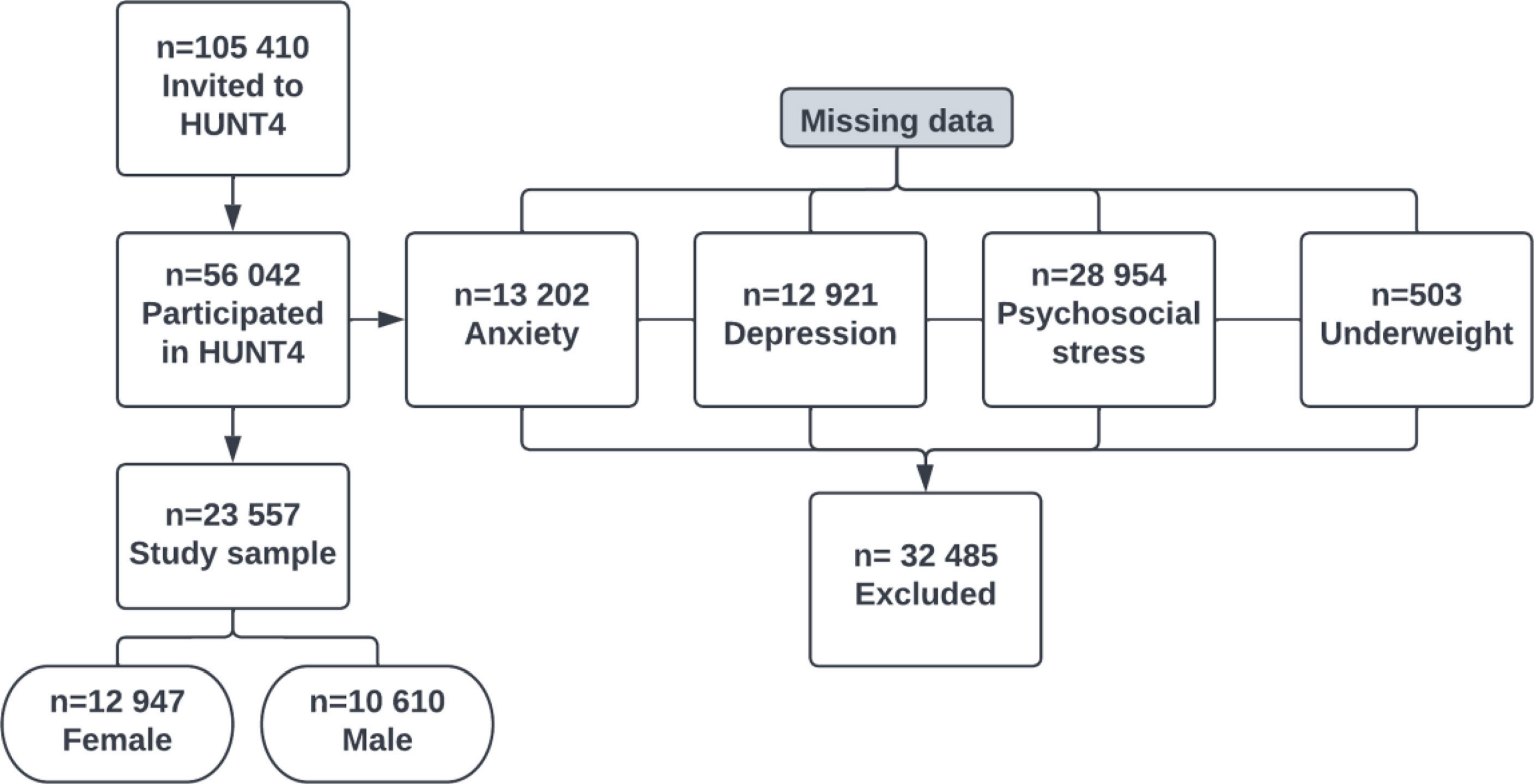
Flowchart of the study population.

The Health Study of Trøndelag (The HUNT Study) is a health survey based on primarily the northern part of the Trøndelag County (23 municipalities) in central Norway (42) where all inhabitants 20 years and above have been invited in the adult part at the estimated time of survey participation. The study consists of four surveys where data have been collected every 11th year: HUNT1 (1984–86), HUNT2 (1995–97), HUNT3 (2006–08) and HUNT4 (2017–19). Cross-sectional data from the fourth wave of the population based Trøndelag Health Study (HUNT4) was used for this study (42). Out of the 103 800 invitees to invited to HUNT4, 56 042 (54.0%) participated. HUNT 4 consisted of biological sampling in addition to a short interview, a clinical examination, and questionnaires which was conducted by trained health professionals at examination stations in each of 23 municipalities in Nord-Trøndelag. In this study, we removed participants with missing data on anxiety (*n* = 13 202), depression (*n* =12 921), psychosocial stress (*n* = 28 945), and participants with underweight (*n* = 503), resulting in 23 557 remaining participants (**Figure 1**). The HUNT Study was approved by the Norwegian Data Inspectorate and Directorate of Health. All participants provided signed, informed consent. Data collection procedures were implemented in accordance with the Helsinki Declaration of 1964, and its later amendments (43). General information about the HUNT4 Survey data is available from the HUNT webpage. The data used for this study was approved by the HUNT Data Access Committee.

### 2.2 Demographic characteristics

Characteristics of the study population including age, sex, marital status, income, and educational attainment was collected. Categorical response options are provided in **Table 1**.

**Table 1:** Characteristics of the study population from the HUNT4 survey in Norway.

Variable	<65 years Adults(N=15896)	≥ 65 years Elderly(N=7154)	Total sample≥20 years old(N=23557)	Variable	<65 years Adults(N=15896)	≥ 65 years Elderly(N=7154)	Total sample≥20 years old(N=23557)
**Gender**	**N (%)**			**BMI**	**N (%)**		
Female	9136 (57.5)	3432 (48)	12947 (55.0)	Normal	5752 (36.2)	2068 (28.9)	8023 (34.1)
Male	6760 (42.5)	3722 (52)	10610 (45.0)	Overweight	6374 (40.1)	3370 (47.1)	9952 (42.2)
**Age at participation**				Obesity class I	3529 (22.2)	1659 (23.2)	5273 (22.4)
20-39 years			5867 (24.9)	Obesity class II & III	241 (1.5)	57 (0.8)	309 (1.3)
40-59 years			7906 (33.6)	**Depression score**	**HADS-D**		
60-79 years			8597 (36.5)	Normal	14665 (90.8)	6703 (90.6)	19405 (82.4)
80-99 years			1187 (5.0)	Mild	1070 (6.6)	562 (7.6)	2687 (11.4)
**Marital status**				Moderate	362 (2.2)	126 (1.7)	1212 (5.1)
Unmarried	6682 (42)	319 (4.5)	7150 (30.4)	Severe	60 (0.4)	9 (0.1)	253 (1.1)
Married	7662 (48.2)	5139 (71.8)	13029 (55.4)	**Anxiety score**	**HADS-A**		
Widow(er)	104 (0.7)	935 (13.1)	1092 (4.6)	Normal	12884 (79.7)	6521 (88.1)	21368 (90.7)
Divorced	1252 (7.9)	725 (10.1)	2005 (8.5)	Mild	2044 (12.7)	643 (8.7)	1632 (6.9)
Separated	196 (1.2)	36 (0.5)	235 (1.0)	Moderate	1003 (6.2)	209 (2.8)	488 (2.1)
**Income per annum (NOK)**				Severe	226 (1.4)	27 (0.4)	69 (0.3)
Less than 250000	1036 (6.5)	977 (13.7)	2026 (8.8)	**Psychosocial stressors**	**Total**		
Between 250000 and 450000	2227 (14.0)	2743 (38.3)	4980 (21.6)	No stressors	3236 (20.0)	2477 (33.5)	5713 (24.3)
Between 450000 and 750000	4404 (27.7)	2497 (34.9)	6912 (29.9)	1-3 stressors	9757 (60.4)	4411 (59.6)	14168 (60.1)
Between 750000 and 1000000	4396 (27.7)	643 (9.0)	5047 (21.9)	4-7 stressors	2994 (18.5)	505 (6.8)	3499 (14.9)
More than 1000000	3833 (24.1)	294 (4.1)	4130 (17.9)	8-10 stressors	170 (1.1)	7 (0.1)	177 (0.8)
**Education**				**Psychosocial stressors**	**Childhood & adolescence**		
9-10 years schooling	683 (4.3)	1755 (24.5)	2558 (10.9)	No stressors	8757 (54.2)	5650 (76.4)	14407 (61.2)
1-2 years academic/vocational training	1807 (11.4)	1710 (23.9)	3629 (15.4)	1-3 stressors	6710 (41.5)	1704 (23.0)	8414 (35.7)
3 years academic/vocational training	2284 (14.4)	509 (7.1)	2879 (12.2)	4-7 stressors	679 (4.2)	46 (0.6)	725 (3.1)
4 years academic/vocational training	3720 (23.4)	1275 (17.8)	5082 (21.6)	8-10 stressors	11 (0.1)	–	11 (0.0)
3 years college/university	3756 (23.6)	1063 (14.9)	4887 (20.7)				
4+ years college/university	3646 (22.9)	842 (11.8)	4522 (19.2)				

Bold values indicates the different age groups in the study sample.

### 2.3 Assessments and outcome measures

#### 2.3.1 Hospital anxiety and depression scale

The HADS is a 14-item self-report questionnaire translated into Norwegian by a psychiatric research group from HUNT2 (44). An anxiety score (HADS-A) and a depression score (HADS-D) can be summed to measure symptoms of anxiety and depression, respectively, or combined to measure symptoms of both anxiety and depression (HADS-total). Response options range from 0 (*not at all*) to 3 (*nearly all the time*). For each subscale, scores are categorised as severe (15–21), moderate (11–14), mild (8–10), and normal (< 8). Validation studies have demonstrated high sensitivity and specificity for scores ≥ 8 for both subscales (45, 46). The Norwegian HADS translation has been validated for populations between 18 and 79 years old with an adequate average internal consistency for the depression (Cronbach’s α = .75) and anxiety (Cronbach’s α = .80) subscales (47). In the regression models, HADS-A (anxiety) and HADS-D (depression) were included as continuous sum scores.

#### 2.3.2 Psychosocial stress

**Figure 2:**
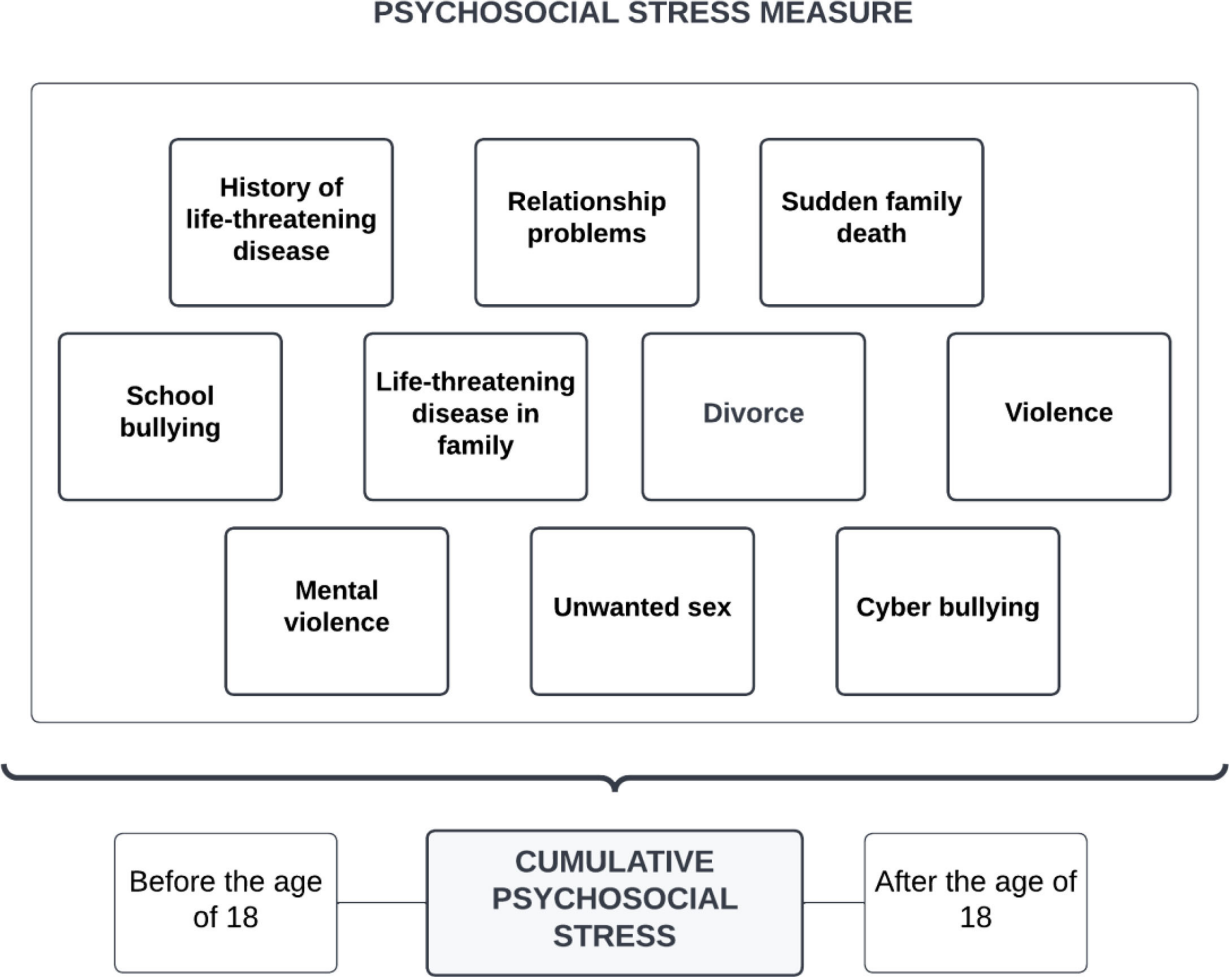
Variables included in the psychosocial stress measure.

In this study, we used a self-report measure of psychosocial stress including potentially traumatic events. The measure of potentially traumatic events was originally used in the Tromsø VII Survey, conducted by (UiT) The Arctic University of Norway (2015–2016) (48). We modified this self-report measure for the current study to capture additional domains of psychosocial stress. Thus, the psychosocial stress screen from the HUNT4 Survey (**Figure 2**) had 10 questions regarding a wide range of psychosocial stress, including 1) a history of life-threatening disease, 2) life-threatening disease in family, 3) relationship problems, 4) divorce, 5) sudden family death, 6) violence, 7) mental violence, 8) unwanted sex, 9) cyber bullying, and 10) school bullying. The 10 items were scored on a categorical scale (yes/no). The final score of the yes responses were aggregated into a cumulative measure of psychosocial stress. The psychosocial stress screen also measured if the exposure occurred in childhood/adolescence (before the age of 18) or in adulthood (after the age of 18).

#### 2.3.3 Obesity classification

Obesity was classified using BMI, measured by weight (kilograms) divided by height squared (metres) (1). Height and weight were measured with the participants wearing light clothes and no shoes by trained health professionals at examination stations. Height was measured to the nearest centimetre (cm) and weight to the nearest half kilogram (kg) (49). We adopted the World Health Organisation’s (WHO) classifications for BMI, including 18.5 to 24.9 kg/m^2^ (normal range), 25 to 29.9 kg/m^2^ (overweight), 30 to 34.9 kg/m^2^ (obesity class I), 35 to 39.9 kg/m^2^ (obesity class II) and ≥ 40 kg/m^2^ (obesity class III). Height (cm) and weight (kg) were measured using bioelectrical impedance (InBody 770, Cerritos, CA, USA).

### 2.4 Data analysis

Obesity classes II and III were combined in analyses for brevity and because of the limited number of participants in each group. Prior to conducting the multivariable models, we tested for multicollinearity among covariates where the highest variance of inflation was <1.5, hence no indication of multicollinearity was found. We divided the sample into adults (<65 years old), (N=15896) and elderly (≥ 65 years old), (N=7154) and performed analyses for each age cohort. Initially, three regression models were run exploring the association between BMI categories and either depression (HADS-D) or anxiety (HADS-A), and both combined (HADS-total), adjusting for the covariates sex, age, educational attainment, marital status and income. Further, two models were run investigating the association between BMI categories and psychosocial stress adjusting for sex, age, and educational attainment (Model 1), and the association between BMI categories and psychosocial stress where anxiety and depression combined (HADS-total), sex, age, educational attainment, marital status and income were included (Full Model). Association analyses that explored the association between BMI categories and psychosocial stress where anxiety and depression combined (HADS-total), sex, age, educational attainment, marital status, and income were included, including life course exposure to psychosocial stress or exposure in childhood and adolescence (before the age of 18) (**Table 2**). Descriptive statistics were explored using SPSS Statistics Version 26.

## 3 Results

The final sample was 55% female and ranged in age between 20 to 99.6 years (*M* = 53.8, *SD* = 17.5), with an average BMI of 27.2 (*SD* = 4.6). The most common income range was 451 000–750 000 NOK (29.9%), the most common educational level was 3–4 years of vocational school or apprenticeship (21.6%), and 55.4% of the participants were married. See **Table 1** for descriptive statistics. In total, 17.6% of all participants reported symptoms of depression, while 9.3% reported symptoms of anxiety (**Table 1**). There was a positive correlation between HADS-A and HADS-D, N=23557, *r*=.552, p <.001.

A statistically significant relationship was found between depression and overweight (OR = 1.02, 95% CI [1.01, 1.04], *p* <.001), obesity I (OR = 1.05, 95% CI [1.03, 1.06], *p* <.001) and obesity II/III (OR = 1.10, 95% CI [1.06, 1.14], *p* <.001), among the adult participants, adjusting for sex, age, educational attainment, marital status, and income (see **Table 3**). The positive relationship between BMI and higher symptoms of depression increased in magnitude as symptoms of depression increased. No statistically significant relationship between depression and higher levels of BMI was found, after controlling for covariates among the elderly participants (**Table 3**).

**Table 3:** Odds of BMI category considering symptoms of depression and anxiety.

		Age < 65 years			Age ≥ 65 years		
	BMI category	Odds (95% CI)	SE	P-value	Odds (95% CI)	SE	P-value
**Model 1**							
**Depression** **(HADS-D)**	Overweight	1.02 (1.01, 1.04)	0	<.001	0.98 (0.96, 1.00)	0.01	.099
	Obese class I	1.06 (1.05, 1.08)	0.01	<.001	1.02 (0.99, 1.04)	0.01	.143
	Obese class II and III	1.12 (1.07, 1.16)	0.02	<.001	1.04 (0.96, 1.14)	0.05	.342
**Model 2**							
**Anxiety** **(HADS-A)**	Overweight	1.00 (0.99, 1.01)	0.01	.723	0.97 (0.95, 0.99)	0.01	.002
	Obese class I	1.01 (0.99, 1.02)	0.01	.344	0.97 (0.95, 0.99)	0.01	.002
	Obese class II and III	1.03 (0.99, 1.06)	0.02	.163	1.01 (0.93, 1.09)	0.04	.898
**Model 3**							
**HADS-total**	Overweight	1.01 (1.00, 1.01)	0.01	.07	0.99 (0.97, 1.00)	0.01	.006
	Obese class I	1.02 (1.01, 1.03)	0.00	<.001	0.99 (0.98, 1.01)	0.01	.300
	Obese class II and III	1.04 (1.02, 1.06)	0.01	<.001	1.01 (0.97, 1.06)	0.02	.325

Model 1 considers depression (HADS-D) adjusted for sex, age, educational attainment, marital status, and income.

Model 2 considers anxiety (HADS-A), adjusted for sex, age, educational attainment, marital status, and income.

Model 3 considers anxiety and depression (HADS total), adjusted for sex, age, educational attainment, marital status, and income.

Among the adults, no statistically significant relationship between anxiety and higher levels of BMI was found, after controlling for covariates (see **Table 3**). For the elderly, a statistically significant relationship was found between anxiety and overweight (OR = 0.97, 95% CI [0.95, 0.99], *p* = .002), and obesity I (OR = [0.95, 0.99], *p* = .002) adjusting for sex, age, educational attainment, marital status, and income.

In the model, for the adult participants, considering anxiety and depression combined (HADS-total), the association with anxiety and depression was statistically significant with both obesity class I (OR = 1.02, 95% CI [1.01, 1.03], *p* < .001) and obesity II and III (OR = 1.04, 95% CI [1.02, 1.06], *p* <.001), but not for those with overweight (OR = 1.01, 95% CI [1.00, 1.01], *p* = .070). Among the elderly, the association with anxiety and depression was statistically significant with overweight (OR=0.99, 95% CI [0.97, 1.00], *p* = .006) but not with any of the obesity classes (see **Table 3**).

The majority (60.1%) of the participants reported having experienced 1-3 stressors, while 15.6% reported 4-10 stressors during their life course (**Table 1**). Psychosocial stress significantly and positively related to all levels of BMI, with or without considering anxiety and depression, after controlling for covariates among all participants (**Tables 2**, **4**). Among the adult participants, a positive relationship was found between exposure to psychosocial stress both in childhood/adolescence and through the life course with overweight and all obesity classes without considering anxiety and depression (**Table 2**). The strongest relationship was found between exposure to psychosocial stress in childhood/adolescence and obesity class II and III without the HADS-total being included in the model (OR = 2.12, 95% CI [1.72, 2.61], *p* <.001), and when considering HADS-total (OR = 2.01, 95% CI [1.62, 2.49], *p* <.001) among participants <65 years old. The relationships showed the same trend for exposure to life course stressors with slightly weaker effects in the highest weight categories. For every one-unit of increase in number of psychosocial stressors, the odds of falling into a higher category of BMI increased. For example, among the adults, for every one-unit increase of psychosocial stressors in childhood, the odds of falling into the obesity class I category increased by 17.3%, while the odds of falling into the obesity class II and III increased by 34.4%, without adjusting for anxiety and depression (**Table 2**). Similar, but weaker associations between psychosocial stress and BMI were found among the elderly, with the exception of exposure to psychosocial stress in childhood/adolescence and overweight (**Table 4**). Adjusting for anxiety and depression did not change the association between psychosocial stress and BMI and was consistent across all three BMI categories in the full regression models in both age groups compared to normal weight (**Tables 2**, **4**).

**Table 2:** Odds of BMI category considering psychosocial stressors over life course and in childhood/adolescence* adjusted for symptoms of anxiety and depression combined (adults).

Participants(<65 years)		Life course			Before 18 yrs.		
	BMI category	Odds (95% CI)	SE	P-value	Odds (95% CI)	SE	P-value
**Model 1**							
Psychosocial stressors	Overweight	1.17 (1.11, 1.24)	0.03	< .001	1.15 (1.07, 1.23)	0.03	< .001
	Obese class I	1.37 (1.28, 1.47)	0.04	< .001	1.39 (1.29, 1.50)	0.04	< .001
	Obese class II and III	1.73 (1.42, 2.10)	0.10	< .001	2.12 (1.72, 2.61)	0.11	< .001
**Full model**							
Psychosocial stressors	Overweight	1.17 (1.10, 1.24)	0.03	< .001	1.14 (1.07, 1.22)	0.03	< .001
	Obese class I	1.34 (1.25, 1.44)	0.04	< .001	1.36 (1.26, 1.47)	0.04	< .001
	Obese class II and III	1.62 (1.32, 1.98)	1.03	< .001	2.01 (1.62, 2.49)	0.11	< .001

Defined as before 18 years old. Model 1 considers adjusted for sex, age, and educational attainment. Full Model considers psychosocial stress adjusted for depression and anxiety (HADS-total), sex, age, educational attainment, marital status, and income.

**Table 4:** Odds of BMI category considering psychosocial stressors over life course and in childhood/adolescence* adjusted for symptoms of anxiety and depression (elderly).

Participants (≥ 65 years)		Life course			Before 18 yrs		
	BMI category	Odds (95% CI)	SE	P-value	Odds (95% CI)	SE	P-value
**Model 1**							
Psychosocial stressors	Overweight	1.13 (1.02, 1.25)	0.05	.018	1.11 (0.97, 1.26)	0.07	.119
	Obese class I	1.20 (1.07, 1.35)	0.06	.002	1.17 (1.01, 1.36)	0.08	.041
	Obese class II and III	1.88 (1.17, 3.04)	0.25	.010	1.95 (1.16, 3.28)	0.03	.011
**Full model**							
Psychosocial stressors	Overweight	1.16 (1.04, 1.28)	0.05	.005	1.13 (1.00, 1.29)	0.07	.058
	Obese class I	1.22 (1.08, 1.37)	0.06	.001	1.18 (1.02, 1.37)	0.08	.029
	Obese class II and III	1.86 (1.14, 3.03)	0.25	.012	1.92 (1.14, 3.25)	0.27	.015

Defined as before 18 years old Model 1 considers adjusted for sex, age, and educational attainment. Full Model considers psychosocial stress adjusted for depression and anxiety (HADS-total), sex, age, educational attainment, marital status, and income.

## 4 Discussion

This study aimed to explore how depression, anxiety, and psychosocial stress individually relate to BMI across four weight categories: normal weight, overweight, obesity class I, and obesity classes II and III. Firstly, as hypothesised, results indicated that for every unit increase in depression, the odds of falling into a higher BMI class significantly increased after controlling for sex, age, educational attainment, marital status, and income. Secondly, anxiety was associated with overweight and obesity class I among the elderly participants. Thirdly, as hypothesised, after controlling for covariates, for every unit increase in number of psychosocial stressors, both life course and during childhood/adolescence, the odds of falling into obesity classes I, II and III increased significantly. This result remained consistent after controlling for depression and anxiety combined.

Previous research has demonstrated that as depression increases, so does BMI (10), which is supported by our results even after controlling for the potentially comorbid influences of anxiety and psychosocial stress. In addition, depression and obesity remained significantly and positively related, while controlling for the effect of sex, age, educational attainment, marital status, and income among participants <65 years. In contrast, however, Choi and colleagues (16) found a negative association between depression and BMI in a community sample with low socioeconomic status. This discrepancy may be because we controlled for educational attainment, which is often used as a proxy of socioeconomic status. Further, the propensity for individuals with depression to be sedentary may perpetuate both obesity and depression (50). For example, studies have shown an association between physical activity, depression, and obesity (50). As a result, this may increase the complexity of treating obesity independently of depression.

In contrast to previous research (9), our results suggested that anxiety was not positively associated with obesity among the adult participants. Statistically significant findings between anxiety and higher levels of BMI were, however, found among the elderly participants while controlling for multiple variables, potentially providing a more accurate relationship between anxiety and BMI by stratifying the age groups. Importantly, previous studies contain methodological limitations and weak associations (9). For instance, previous research is limited by poor confounder control (9, 19). Our results showing an association between anxiety and higher BMI among the elderly, may be due to the relationship between the body’s stress response system (HPA axis) (51) and anxiety-reducing behaviours (e.g., emotional eating) (52). Binge eating behavior is commonly associated with elevated body weight, and disordered eating patterns such as binge eating may lead to a temporary reduction in anxiety. Previous studies have found that anxiety is a stronger predictor of binge eating disorder (BED) than depression (53), and severe cases of binge eating can result in a clinical diagnosis of BED, which is characterised by greater preoccupations with food, poor dietary restraint, body dissatisfaction, psychological distress, and low self-esteem (53, 54). As anxiety disorders are commonly seen in individuals with BED (55), there is a need for further research to elucidate the temporal associations between BED, weight problems and anxiety.

Consistent with previous literature (28, 32), we found that as the number of past psychosocial stressors increases, so does the odds of falling into a higher BMI class. Even after controlling for the potentially confounding variables of depression, anxiety, sex, age, educational attainment, marital status, and income, this association remained consistent. Thus, to improve outcomes of lifestyle interventions, health care providers could incorporate routine screening of past and current psychosocial stressors experienced by a patient. Furthermore, psychosocial stress may contribute to the development of disordered eating behaviours such as binge eating that act as self-regulatory coping strategies, potentially contributing to difficulties with long-term weight management. Therefore, obesity treatment programs could incorporate psychological intervention to address psychosocial stress and replace maladaptive self-regulatory strategies with more adaptive coping methods. This holistic treatment approach may improve long-term weight maintenance following lifestyle intervention. Further research is required to assess the efficacy of incorporating treatment targeting psychosocial stress in obesity interventions.

Although not a primary focus of this study, there were notable results regarding the relationship between sex and BMI that would be relevant to explore in future research. Across all regression models, males were significantly more likely to be overweight than females; however, females were more likely to be classified in the obesity categories compared to males. These findings are supported across current literature (56, 57).

In addition, similar to previous research, our study showed that individuals with higher levels of education were less likely to be overweight and have obesity than individuals with less education (37). Future studies exploring mechanisms related to various domains of adversity, education, and the risk of obesity, may influence and improve prevention and treatment of obesity.

### 4.1 Limitations and strengths

The findings of this study should be considered in the context of several limitations. Firstly, the sample data from this study was exclusively derived from Norway, which has a welfare model different from many other countries. Therefore, while conclusions from this study can be generalised to individuals from Norway, they may not equally generalise to other populations. Secondly, this study employed self-report measures to operationalise depression, anxiety, and psychosocial stress. Research suggests that individuals often misestimate their experiences when self-reporting, whereas clinical interviews represent a ‘gold standard’ assessment method (58). Furthermore, assessing current anxiety symptoms compared to a history of anxiety (20), may make comparisons to other studies less reliable. Additionally, anxiety may be measured through self-report measures or structured/semi-structured interview, with some researchers targeting a specific anxiety disorder (e.g., social phobia), while others measuring general anxiety symptomology. These methodological differences may contribute to variations in results. Finally, we cannot definitively establish temporality between exposure to psychosocial stress and body weight. However, our study illustrates that body weight may be modifiable by exposure to psychosocial stressors which may in the future be studied to determine causal relationships.

Limitations notwithstanding, there are several important strengths in this study. Firstly, the sample size was large, allowing us to stratify adults and elderly, and included a wide range of demographic information, which enabled confounding variables to be included in the analyses. The dataset also had a relatively equal distribution between males and females, which is uncommon in obesity studies. Secondly, other than underweight, the study covered all weight classes, which increases the external validity of these results to most individuals in the general population of Norway. Thirdly, our robust statistical analysis and consideration of confounding variables enables a comprehensive examination of the variables of interest (i.e., depression, anxiety, and psychosocial stress), while controlling for potentially confounding variables. Collectively, these strengths increase the reliability and validity of the findings of this study.

### 4.2 Clinical implications and future directions

The findings from this study highlight that anxiety, depression and psychosocial stress may play an important role in obesity maintenance and may potentially interfere with maintaining a healthy weight or preventing weight gain. This emphasises the importance of assessing anxiety and depression, and if present, developing targeted treatments within a larger obesity program. In support of this approach, previous literature has found that depression treatment was effective for obtaining weight loss, compared to a control group (59).

More, assessment of anxiety and depression and psychosocial stress including past traumatic events may improve obesity treatments. If undetected, these psychological difficulties may hinder the understanding of the patient’s behaviours related to diet and exercise, and thus the successful course of treatment. A better understanding of patients’ past and current psychosocial stress may increase treatment compliance. In addition, integrating evidence-based psychological interventions for depression, anxiety, and psychosocial stress should be a necessary component in obesity treatments. If untreated, psychopathology may impede weight loss and weight maintenance.

Overall, obesity interventions which considers the negative health outcomes associated with psychosocial stress, may successfully improve weight loss and weight loss maintenance for individuals with obesity and comorbid mental health problems. Future research should also seek to explore and clarify the relationship between anxiety and obesity with a consistent conceptualisation of anxiety. Further, due to the variability in psychosocial stress, future studies should distinguish which types of stress have better predictive effect on the development of obesity. More, time of exposure to psychosocial stress needs to be better understood, while controlling for potentially comorbid disorders such as depression and anxiety. Undoubtedly, this will improve accurate assessment and treatment of obesity in light of patient adversities. In addition, it is important to explore the impact of stressors manifested through body image disorders and eating disorders. By understanding mechanisms concerning how psychosocial stress interacts with weight development and management in obesity, targeted interventions could be provided following adverse events to minimize the detrimental mental and physical health outcomes.

## 5 Conclusion

Substantial evidence is accumulating that identifies obesity as a multifaceted health problem. This study has shown that anxiety, depression and psychosocial stress are independently associated with BMI. Future research should continue to explore the complex nature of obesity and develop more appropriate assessments and interventions.

## Data availability statement

Publicly available datasets were analyzed in this study. Data used from the HUNT survey in research projects will, when reasonably requested by others, be made available on request to the HUNT Data Access Committee (hunt@medisin.ntnu.no). The HUNT data access information (available here: http://www.ntnu.edu/hunt/data) describes in detail the policy regarding data availability.

## Ethics statement

The studies involving human participants were reviewed and approved by Regional Committee for Medical Research Ethics Central Norway. The patients/participants provided their written informed consent to participate in this study.

## Author contributions

TE-N contributed with design of the study, wrote the final manuscript and prepared data analysis and interpretation with KK. AT wrote the first draft of the manuscript, and provided critical feedback on the final manuscript. DS contributed to the study by providing critical feedback and helped shape the final manuscript. JR contributed to the study by conceiving and designing the study, and provided critical feedback and helped shape the final manuscript. KK contributed to the study by conceiving and designing the study, arranged material preparation and secondary data collection. All authors contributed to the article and approved the submitted version.

## Acknowledgments

The Trøndelag Health Study (HUNT) is a collaboration between HUNT Research Centre (Faculty of Medicine and Health Sciences, Norwegian University of Science and Technology NTNU), Trøndelag County Council, Central Norway Regional Health Authority, and the Norwegian Institute of Public Health. Our sincere appreciation to HUNT4 participants and HUNT researchers for providing valuable data and enabling the progression of knowledge in this important field.

## Conflict of interest

The authors declare that the research was conducted in the absence of any commercial or financial relationships that could be construed as a potential conflict of interest.

## Publisher’s note

All claims expressed in this article are solely those of the authors and do not necessarily represent those of their affiliated organizations, or those of the publisher, the editors and the reviewers. Any product that may be evaluated in this article, or claim that may be made by its manufacturer, is not guaranteed or endorsed by the publisher.
